# Extending the Overnight Fast: Sex Differences in Acute Metabolic Responses to Breakfast

**DOI:** 10.3390/nu12082173

**Published:** 2020-07-22

**Authors:** Fiona S. Atkinson, Gabriella A. Heruc, Verena M. H. Tan, Peter Petocz, Jennie C. Brand-Miller

**Affiliations:** 1 Charles Perkins Centre and School of Life and Environmental Sciences, The University of Sydney, Sydney 2006, Australia; fiona.atkinson@sydney.edu.au (F.S.A.); g.heruc@westernsydney.edu.au (G.A.H.); verena.tan@singaporetech.edu.sg (V.M.H.T.); 2 Department of Mathematics and Statistics, Macquarie University, Sydney 2109, Australia; peter.petocz@mq.edu.au

**Keywords:** glucose tolerance, insulin sensitivity, overnight fasting, time-restricted feeding, postprandial glycaemia

## Abstract

Fasting for over 24 h is associated with worsening glucose tolerance, but the effect of extending the overnight fast period (a form of time-restricted feeding) on acute metabolic responses and insulin sensitivity is unclear. The aim of this pilot study was to determine the acute impact of an increased fasting period on postprandial glycaemia, insulinemia, and acute insulin sensitivity responses to a standard meal. Twenty-four lean, young, healthy adults (12 males, 12 females) consumed a standard breakfast after an overnight fast of 12, 14, and 16 h. Each fast duration was repeated on three separate occasions (3 × 3) in random order. Postprandial glucose and insulin responses were measured at regular intervals over 2 h and quantified as incremental area under the curve (iAUC). Insulin sensitivity was determined by homeostatic modelling assessment (HOMA). After 2 h, ad libitum food intake at a buffet meal was recorded. In females, but not males, insulin sensitivity improved (HOMA%S +35%, *p* = 0.016, marginally significant) with longer fast duration (16 h vs. 12 h), but paradoxically, postprandial glycaemia was higher (glucose iAUC +37%, *p* = 0.002). Overall, males showed no differences in glucose or insulin homeostasis. Both sexes consumed more energy (+28%) at the subsequent meal (16 h vs. 12 h). Delaying the first meal of the day by 4 h by extending the fasting period may have adverse metabolic effects in young, healthy, adult females, but not males.

## 1. Introduction

Regular breakfast consumption is associated with improved weight loss maintenance [1], a decline in impulsive snacking [2], and greater cognitive performance [3]. Despite these benefits, trends since 1965 indicate that breakfast consumption by both adults [4] and children [5] is declining. Conversely, “breakfast skipping” behaviour is associated with adverse health effects, including higher body weight [6,7], a greater risk of obesity [8,9], and an increased risk of type 2 diabetes [10,11]. Breakfast skipping in regular consumers induces higher intakes of energy-dense foods later in the day, increasing overall energy consumption while reducing diet quality [12].

The potential deleterious effects of breakfast skipping are also relevant to eating plans that recommend an increased fasting duration, such as time-restricted feeding [13,14]. From a metabolic perspective, an extended overnight fast of 16–18 h has been associated with lower plasma glucose concentration and a corresponding rise in fatty acid oxidation when compared with a more typical 10–12 h fast [15]. Prolonged fasting duration (72 h or more) leads to greatly elevated postprandial glucose and insulin responses to the next meal [16], along with impaired glucose tolerance [17,18] and insulin-mediated glucose disposal [19]. Whether this is also characteristic of shorter periods of fasting is unclear.

We hypothesised that extending the duration of the overnight fast (i.e., increasing the time-restricted feeding period) in healthy adults may acutely increase postprandial hyperglycaemia, hyperinsulinemia, or both and thereby contribute to the adverse health effects. Higher blood glucose responses to meals, even in individuals with normal glucose tolerance, increase oxidative stress and β-cell insulin secretion [20]. The aim of the present study was to investigate the acute effects of prolonging the overnight fast from 12 to 14 to 16 h on postprandial responses, insulin sensitivity, and subsequent ad libitum energy intake. Improved understanding of metabolic processes occurring in response to short fasting, induced either through time-restricted feeding or the skipping or delaying of breakfast, may help elucidate specific lifestyle behaviours that lead to adverse health effects.

## 2. Materials and Methods

### 2.1. Study Population

Healthy adults, 12 males and 12 females, were recruited from the University of Sydney student population. Smokers and individuals with special dietary requirements were not eligible. Participants were required to maintain their weight, exercise, and eating patterns for the study duration (10 weeks) and to refrain from alcohol and strenuous exercise on the days before testing. The Human Research Ethics Committee of the University of Sydney approved the study protocol (08-2005/7958), and participants gave written, informed consent.

### 2.2. Study Design

In a randomised, within-subject, repeated measures study design, each individual completed nine separate testing sessions in which the fasting period lasted for 12, 14, or 16 h. Each fasting duration was tested three times (3 × 3) by each participant. At 19:00 on the night before testing, participants consumed a standard evening meal provided by the investigators containing 55% energy (E) as carbohydrate, 15% E as protein, and 30% E as fat. The food components in the evening meal were constant, but absolute E intake varied according to participant sex (2745 kJ for males and 2120 kJ for females). Participants were instructed not to eat or drink, apart from water, prior to the test meal given at breakfast in the metabolic kitchen at 7:00, 9:00, or 11:00. The standardised breakfast meal, consumed within 12 min during each test session, consisted of 34 g flaked corn cereal with 150 mL low-fat milk, 36 g white bread with 10 g strawberry jam, and a small glass (190 mL) of orange juice. The test meal contained 1645 kJ, 75 g available carbohydrate, 3 g fat, 12 g protein, and 3 g fibre. Baseline finger-prick capillary blood samples were taken at −20, −10, and 0 min (fasting) and at 15, 30, 45, 60, 90, and 120 min after the start of the meal. To improve peripheral circulation, participants soaked their hands in hot water for 2 min prior to each blood collection. An automated lancet device (Safe-T-Pro, Boehringer Mannheim GmbH, Mannheim, Germany) was used to collect ~0.7 mL of blood. At each timepoint, participants rated their feelings of satiety on a validated 7-point Likert scale [21]. After the final blood sample, participants were free to consume food and drink ad libitum from a selection of common foods (breads, cereals, spreads, cold cuts, cheese, yoghurt, milk, juice, coffee, tea, eggs, fruit, biscuits, and crisps) but were required to weigh and record the amounts eaten using digital scales.

Blood was collected into heparin-coated Eppendorf tubes (10 IU heparin sodium salt, Sigma Chemical Co., St. Louis, MO, USA) and centrifuged at 12,000 × *g* for 60 s. Plasma was collected in uncoated tubes and stored at −20 °C for later analysis. Plasma glucose was assayed in duplicate using an automatic spectrophotometric centrifugal analyser (Roche Hitachi 912, Boehringer Mannheim GmbH, Mannheim, Germany) using the hexokinase/glucose-6-phosphate dehydrogenase enzymatic assay. Plasma insulin was assessed using an antibody-coated tube radioimmunoassay kit (Coat-A-Count Insulin, Diagnostic Products, Los Angeles, CA, USA). Subsequent food intake was analysed using FoodWorks Professional (version 8.0, Xyris Software, Brisbane, Australia).

### 2.3. Data Analysis

Results are presented as mean ± SEM. Postprandial plasma glucose and insulin concentrations were assessed as the incremental area under the curve (iAUC) over 120 min calculated according to the trapezoidal rule, with the fasting concentration (average of −20, −10, and 0 min timepoints) as the baseline [22]. Insulin resistance was assessed by the original homeostasis model assessment (HOMA1-IR) formula, i.e., (fasting glucose (mmol/L) × fasting insulin (pmol/L))/22.5 [23], and by computer modelling (HOMA2-IR) [24]. Insulin sensitivity (HOMA %S, the reciprocal of HOMA2-IR) and β-cell (HOMA %B) function were also calculated. Statistical analyses were performed using SPSS for Windows (version 19.0, SPSS, Chicago, IL, USA). Analysis of variance (ANOVA) models were used to compare the effects of fasting period and participants on plasma glucose, insulin, and ad libitum food intake. Time of fasting was included as a fixed factor and subjects as random factors, together with their interaction. When time-by-subject interactions were significant, further model analyses investigated the effects of sex and ethnicity. Post hoc comparisons (with Bonferroni adjustment) were used to examine differences between time-points. Because a large number of comparisons were made, a value of *p* < 0.05 was considered marginally significant and *p* < 0.01 statistically significant.

## 3. Results

### 3.1. Participant Characteristics

The group of 24 healthy adults had a mean age ± SD of 23.0 ± 2.6 years and a mean BMI ± SD of 22.1 ± 2.5. Eleven participants were of European–Caucasian background and 13 of Southeast Asian origin. All participants completed the nine test sessions.

### 3.2. Postprandial Glycaemia, Insulinemia, and Measures of Insulin Sensitivity

In the whole participant group (*n* = 24), fasting plasma glucose and insulin concentrations decreased significantly (*p* = 0.002 and 0.045, respectively) as the duration of fasting increased, with no specific sex differences. Because ANOVA identified a significant effect of sex on several indices of glucose homeostasis, the results are presented separately for females (Table 1) and males (Table 2). In females, but not males, insulin sensitivity as determined by computer modelling of HOMA (%S) increased by 42% as the length of fasting increased from 12 to 16 h (*p* = 0.016, Table 1). HOMA-IR also improved in females only from 12 to 16 h (log-transformed data, *p* = 0.024, Table 1). HOMA %B, a measure of β-cell secretion, was not affected in either sex.

**Figure 1:**
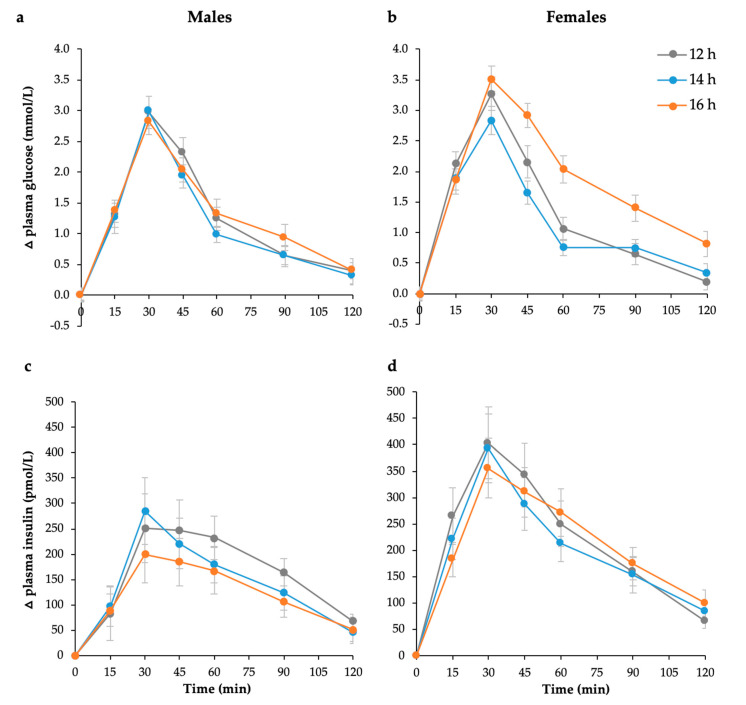
Mean (± SEM) changes in postprandial plasma glucose in (**a**) males (*n* = 12) and (**b**) females (*n* = 12) and postprandial plasma insulin responses in (**c**) males (*n* = 12) and (**d**) females (*n* = 12) to 75 g available carbohydrate standard breakfast at 12, 14, and 16 h fasting periods.

**Figure 2:**
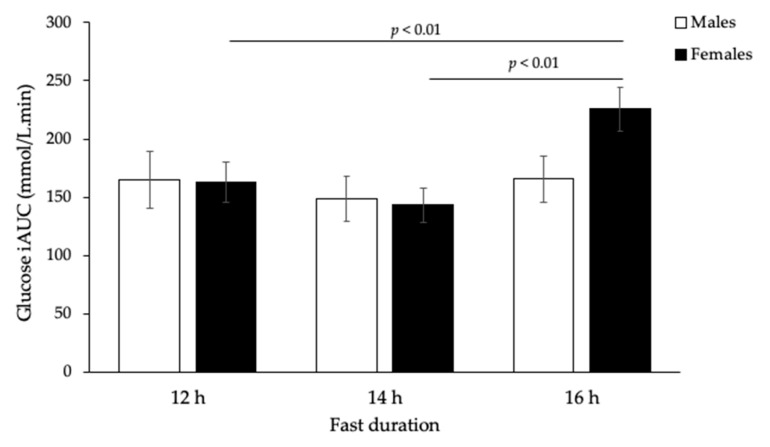
Incremental glucose response (iAUC) after 75 g carbohydrate standard breakfast at 12, 14, and 16 h fasting periods (*n* = 24, *p* < 0.01, two-way ANOVA). There was also an overall significant difference in glucose iAUC between males and females over time (*p* < 0.01, two-way ANOVA).

Postprandial glucose and insulin responses to the 75 g available carbohydrate standard breakfast are shown in Figure 1. Glucose iAUC increased significantly with the duration of overnight fast (*p* = 0.002), but responses were sex-specific (*p* = 0.01, Figure 2). Only females showed a significant increase (*p* < 0.001, Table 1), while males remained unchanged (*p* = 0.326, Table 2). In contrast, insulin iAUC tended to decrease, reaching significance in the males, but not females, after logarithmic transformation (*p* = 0.025, Table 2). The average within-individual CV for glucose iAUC across all nine testing sessions was 30% compared with 24% for a given duration (average of three sessions after the same fast length). Males were less variable than females (21% vs. 29%), but the difference was not statistically significant.

### 3.3. Subsequent Meal Energy Intake

**Figure 3:**
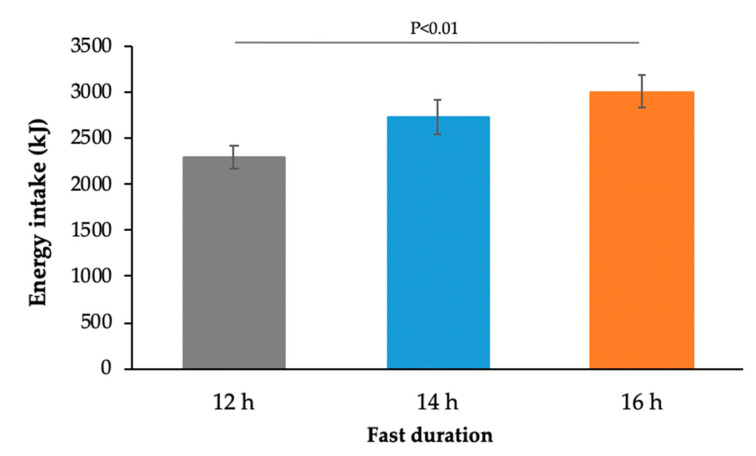
Energy intake at an ad libitum meal consumed 2 h after the standard breakfast meal (12 h: *n* = 59, 14 h: *n* = 64, 16 h: *n* = 67; *p* < 0.01, two-way ANOVA).

Extended fasting produced a significant increase in ad libitum energy intake at the subsequent meal consumed 2 h after the standard breakfast (*p* < 0.001, Figure 3). Protein, total fat, saturated fat, and carbohydrate intake at the subsequent meal all increased significantly with increasing fast duration (*p* < 0.01, Table 3). The effect was more significant in males than females (*p* < 0.05).

## 4. Discussion

In the present study, in young, lean, healthy females, but not males, extending the overnight fast by 4 h improved insulin sensitivity but simultaneously worsened postprandial glycaemia. To our knowledge, this sex-specific effect is a novel finding. The longer but still realistic 16 h fasting period was associated with a clinically important 37% increase in postprandial glycaemic response in females, as assessed by iAUC for a standard breakfast meal. In contrast, males showed no changes that were biologically or statistically significant. These findings are relevant to eating patterns that have extended fasting periods, either due to breakfast skipping behaviour or time-restricted feeding, and to glycaemic index (GI) testing methodology.

Apart from metabolic effects, our study also demonstrated that healthy adults consumed 28% more energy at a subsequent meal following the extended fast (16 h) compared with that following a 12 h overnight fast. There were also corresponding increases in protein, total fat, saturated fat, and total carbohydrate intake at the next meal. These findings are consistent with research suggesting that skipping breakfast is associated with obesity and greater energy intake throughout the remainder of the day [12,25], although not all studies show differences in energy intake [26,27]. It is possible that individuals manage energetic compensation at other times of the day or over the course of a longer timeframe.

We hypothesised that delaying the breakfast meal by extending the overnight fast from 12 to 16 h would impair insulin sensitivity on the basis of previous studies where the fasting duration was considerably longer. Instead, HOMA insulin resistance, a well-accepted measure of hepatic insulin sensitivity [28], appeared to markedly improve when breakfast was delayed by 4 h, at least in females. Fasting periods of 1 to 3 days are known to impair glucose tolerance [16,17,18], but surprisingly, shorter periods less than 24 h have been little studied, despite the relevance to diagnostic testing. Fasting glucose concentration is known to decline with increasing duration of overnight fast, reflecting decreasing glycogen stores and depletion of the carbohydrate-derived energy supply from the last meal [15]. Theoretically, the corresponding decline in insulin concentration promotes the use of fat as a source of fuel and reduced reliance on glucose for energy [29]. Together, the fall in these two biochemical parameters leads to a decrease in HOMA-IR, a product of the fasting glucose and insulin concentration.

HOMA-IR and HOMA%S are known to reflect hepatic rather than peripheral insulin sensitivity. Hence, any improvement in the sensitivity of the liver to insulin would correspond to lower insulin concentrations in the fasting state and higher levels of gluconeogenesis from non-glucose sources, such as glycerol and amino acids [30]. In contrast, the 37% increase in postprandial glycaemia seen in females suggests that peripheral insulin sensitivity declined with extended fasting. The incretin hormone, gastric inhibitory peptide (GIP), may be responsible for this effect. GIP released by K cells, located in the proximal small intestine, has mostly unfavourable metabolic and cardiovascular properties in respect to high-GI meals [31].

The presence of selective insulin resistance in one organ or tissue and not another is well recognised, particularly in the context of obesity, where insulin resistance in muscles may be counterbalanced by insulin sensitivity in adipose tissue stores [32]. Peripheral insulin sensitivity may be a key factor influencing both fasting and postprandial glucose metabolism, which are, in turn, affected by the higher skeletal muscle mass and function in males. We found that while postprandial glucose iAUC increased in females, there was no corresponding increase in the insulin iAUC. Others have also reported that postprandial glucose iAUC increased throughout the day in females but not males without a corresponding change in insulin iAUC [33].

Sexual dimorphism in glucose and lipid metabolism in response to longer periods of fasting (>22–72 h) has been reported [34], with women having greater reliance on lipid metabolism during fasting, hypoglycaemia, and exercise. We can speculate that these sex-specific effects might have an evolutionary basis. Higher oestrogen levels [35] and oestrogen administration itself are known to cause deterioration in glucose tolerance [36]. Females might be metabolically programmed to have exaggerated glucose concentrations after an extended period of fasting in order to enhance survival of the foetus [37,38]. If true, this predisposition could also help to explain the high prevalence of gestational diabetes that develops in otherwise healthy women [37,39].

Our findings are also relevant to GI methodology in which the glucose iAUC after a 50 g available carbohydrate portion of food is compared with that of a reference food [40]. Fasting length may be a source of both intra- and inter-person variability in glucose iAUC response, and therefore could reduce the precision of GI measurement. The International Standards Organisation protocol [41] stipulates a minimum 10–12 h fast but a maximum duration is not specified. Our finding that the intra-individual CV in glucose iAUC was smaller (24% vs. 30%) with the 12 h compared to 16 h fast suggests it is beneficial for participants to maintain a consistent overnight fast duration throughout the course of a study.

Our study has a number of strengths. Each fasting duration treatment was repeated in triplicate to increase within-subject reliability. Fasting concentrations of glucose and insulin were measured at −20, −10, and 0 min before the meal, and therefore HOMA values were calculated using the mean of nine fasting samples (3 tests × 3 time points, −20, −10 and 0 min) [23,42]. We provided the standardised evening meal and used finger-prick sampling, which is more sensitive to acute changes in postprandial glucose than other blood sampling sites [43]. However, the study was not registered as a clinical trial, and the primary and secondary outcomes were not prespecified. Other limitations include the use of an indirect method of assessing insulin sensitivity, which largely reflects hepatic sensitivity. Participants were instructed to avoid excessive alcohol and exercise activity on the day prior to the test, but information on habitual differences was not recorded. Alcohol intake and physical activity are likely to have varied from person to person within the groups, reducing the chances of detecting a difference between the sexes. We did not standardise the phase of the menstrual cycle in women, but this did not compromise the ability to detect differences between men and women. Logarithmic transformation performed on HOMA variables because of skewed distributions also generates a geometric rather than arithmetic mean [42]. Despite providing slightly stronger results overall, presentation of data as geometric means makes results difficult to interpret.

## 5. Conclusions

This pilot study contributes to our understanding of metabolic responses to shorter vs. longer periods of fasting and the potential harmful effects on postprandial glycaemia of delaying the first meal of the day. Given the clinical significance of postprandial hyperglycaemia as a predictor of adverse outcomes [44], the finding that HOMA insulin sensitivity improves in young, lean, and healthy adult females while postprandial glucose deteriorates is novel and worthy of further investigation.

## Figures and Tables

**Table 1:** Plasma glucose and insulin responses for the three overnight fasting periods in females. ^1.^

Variables	12 h	14 h	16 h	*p*-Value ^2^	*p*-Value ^3^
Fasting glucose (mmol/L)	5.2 ± 0.06	5.2 ± 0.06	5.0 ± 0.06	0.058	-
2-h glucose (mmol/L)	5.4 ± 0.10	5.5 ± 0.10	5.8 ± 0.10	0.021 *	-
Glucose iAUC (mmol/L·120 min)	162.9 ± 11.3	143.0 ± 11.3	225.5 ± 11.3	<0.001 **	-
Fasting insulin (pmol/L)	28.0 ± 2.0	24.1 ± 2.0	18.6 ± 2.0	0.056	0.069
2-h insulin (pmol/L)	94.2 ± 11.9	107.8 ± 11.9	118.2 ± 11.9	0.255	0.217
Insulin iAUC (pmol/L·120 min)	26,582 ± 2283	24,176 ± 2283	25,574 ± 2283	0.294	-
Log insulin iAUC (pmol/L·120 min) ^4^	21,456	19,570	21,955	0.342	-
HOMA-IR	0.53 ± 0.03	0.46 ± 0.03	0.35 ± 0.03	0.052	0.064
Log of HOMA-IR ^4^	0.44	0.42	0.32	0.024 *	0.031 *
HOMA (%S)	265.5 ± 19.6	269.9 ± 19.6	359.9 ± 19.6	0.016 *	-
Log of HOMA %S ^4^	226.1	239.8	317.3	0.023 *	0.030 *
HOMA (%B)	55.0 ± 2.2	51.2 ± 2.2	46.3 ± 2.2	0.181	0.205
Log of HOMA %B ^4^	50.4	48.9	44.0	0.254	0.267

^1^ All values are mean ± SEM; *n* = 36 (12 females x 3 repeated tests for each fasting period). iAUC, incremental area under the curve; HOMA-IR, homeostasis modelling assessment of fasting insulin resistance; HOMA (%S), homeostasis modelling assessment of fasting insulin sensitivity; HOMA (%B), homeostasis modelling assessment of fasting β-cell function. ^2^ *p*-values based on two-way ANOVA using a simple model. ^3^ Reanalysed due to significant subject–time interaction in simple model. New *p*-values based on two-way ANOVA using a full factorial model controlling for ethnicity. There was no subject–time interaction in simple model. ^4^ Residual plots of insulin iAUC, HOMA-IR, HOMA %S and HOMA %B suggested logarithmic transformations would yield more accurate results. Log of insulin iAUC, HOMA-IR, HOMA %S and HOMA %B reported as geometric means. SEM is not reported for calculated geometric means. * *p* < 0.05 is marginally significant; ** *p* < 0.01 is statistically significant.

**Table 2:** Plasma glucose and insulin responses for the three overnight fasting periods in males. ^1.^

Variables	12 h	14 h	16 h	*p*-Value ^2^	*p*-Value ^3^
Fasting glucose (mmol/L)	5.4 ± 0.05	5.3 ± 0.05	5.2 ± 0.05	0.024	-
2-h glucose (mmol/L)	5.8 ± 0.11	5.6 ± 0.11	5.6 ± 0.11	0.394	0.410
Glucose iAUC (mmol/L·120 min)	168.8 ± 6.4	149.9 ± 6.4	165.6 ± 6.4	0.326	0.362
Fasting insulin (pmol/L)	24.2 ± 2.1	24.3 ± 2.1	21.2 ± 2.1	0.577	0.450
2-h insulin (pmol/L)	95.2 ± 8.0	78.0 ± 8.0	71.8 ± 8.0	0.239	-
Insulin iAUC (pmol/L·120 min)	21,739 ± 2481	20,255 ± 2481	16,303 ± 2481	0.053	0.080
Log insulin iAUC (pmol/L·120 min) ^4^	15,408	15,287	12,642	0.025	-
HOMA-IR	0.47 ± 0.029	0.46 ± 0.029	0.40 ± 0.029	0.529	0.390
Log of HOMA-IR ^4^	0.40	0.39	0.33	0.232	0.107
HOMA (%S)	289.5 ± 30.0	320.8 ± 30.0	375.8 ± 30.0	0.100	-
Log of HOMA %S ^4^	250.1	258.5	307.0	0.231	0.107
HOMA (%B)	46.7 ± 2.0	47.9 ± 2.0	46.3 ± 2.0	0.941	0.951
Log of HOMA %B ^4^	43.7	43.6	41.4	0.759	0.670

^1^ Values are mean ± SEM; *n* = 36 (12 males x 3 repeated tests for each fasting period). iAUC, incremental area under the curve; HOMA-IR, homeostasis modelling assessment of fasting insulin resistance; HOMA (%S), homeostasis modelling assessment of fasting insulin sensitivity; HOMA (%B), homeostasis modelling assessment of fasting β-cell function. ^2^ *p*-value based on two-way ANOVA using a simple model. ^3^ Two-way ANOVA using a full factorial model controlling for ethnicity. There was no subject–time interaction in simple model. ^4^ Residual plots of insulin iAUC, HOMA-IR, HOMA %S, and HOMA %B indicated a skewed distribution. Log of incremental insulin iAUC, HOMA-IR, HOMA %S, and HOMA %B reported as geometric means. SEM is not reported for calculated geometric means.

**Table 3:** Dietary analysis of ad libitum food intake 2 h after the standard breakfast meal. ^1.^

Variables	12 h	14 h	16 h	*p*-Value ^2^
Energy intake (kJ)	2293 ± 96	2700 ± 91	2936 ± 88	<0.001 **
Protein (g)	18.1 ± 1.3	24.4 ± 1.3	27.9 ± 1.2	0.002 **
Total fat (g)	17.8 ± 1.2	24.8 ± 1.2	27.1 ± 1.2	<0.001 **
Saturated fat (g)	7.1 ± 0.7	9.9 ± 0.6	11.5 ± 0.6	0.001 **
Carbohydrates—total (g)	74.4 ± 3.4	78.4 ± 3.2	83.8 ± 3.1	0.038 *
Carbohydrates—sugars (g)	34.3 ± 2.3	34.9 ± 2.2	36.7 ± 2.2	0.41

^1^ Values are mean ± SEM, only participants who consumed food at the subsequent meal are shown above: *n* = 59 for 12 h, *n* = 64 for 14 h, *n* = 67 for 16 h. ^2^ *p*-values based on two-way ANOVA using a simple model; * *p* < 0.05 is marginally significant; ** *p* < 0.01 is statistically significant.
